# Research progress on repositioning drugs and specific therapeutic drugs for SARS-CoV-2

**DOI:** 10.4155/fmc-2020-0158

**Published:** 2020-07-08

**Authors:** Shiyong Fan, Dian Xiao, Yanming Wang, Lianqi Liu, Xinbo Zhou, Wu Zhong

**Affiliations:** ^1^National Engineering Research Center For The Emergency Drug, Beijing Institute of Pharmacology & Toxicology, Beijing, 100850, China

**Keywords:** clinical trials, COVID-19, repositioning antiviral drugs, SARS-CoV-2, specific antiviral drugs

## Abstract

SARS-CoV-2 has been widely spread around the world and COVID-19 was declared a global pandemic by the World Health Organization. Limited clinically effective antiviral drugs are available now. The development of anti-SARS-CoV-2 drugs has become an urgent work worldwide. At present, potential therapeutic targets and drugs for SARS-CoV-2 are continuously reported, and many repositioning drugs are undergoing extensive clinical research, including remdesivir and chloroquine. On the other hand, structures of many important viral target proteins and host target proteins, including that of RdRp and Mpro were constantly reported, which greatly promoted structure-based drug design. This paper summarizes the current research progress and challenges in the development of anti-SARS-CoV-2 drugs, and proposes novel short-term and long-term drug research strategies.

In December 2019, a cluster of acute respiratory illness cases caused by a new strain of coronavirus was reported. On 11 February 2020, the International Committee on Taxonomy of Viruses (ICTV) officially designates the new coronavirus as SARS-CoV-2; on the same day, the World Health Organization (WHO) named the illness as COVID-19 [1]. COVID-19 was declared a public health emergency of international concern on 30 January 2020 and a global pandemic on 11 March 2020 by WHO. By 27 April 2020, the number of patients infected with SARS-CoV-2 has exceeded 4,000,000 globally, and more than 200,000 have now died of COVID-19.

So far, there is no effective treatment for COVID-19, and the development of anti-SARS-CoV-2 drugs has become an urgent work. In this review, we provide an overview of published scientific information about small molecular anti-SARS-CoV-2 drugs with an emphasis on repositioning antiviral drugs and the novel SARS-CoV-2 specific antiviral drugs for COVID-19. This paper also reviews the challenges in the development of anti-SARS-CoV-2 drugs and proposes short-term and long-term drug research strategies to provide new insights in developing anti-SARS-CoV-2 drugs and repurposing the marketed drugs for COVID-19.

## Structure of SARS-CoV-2 & potential drug targets

**Figure 1.**
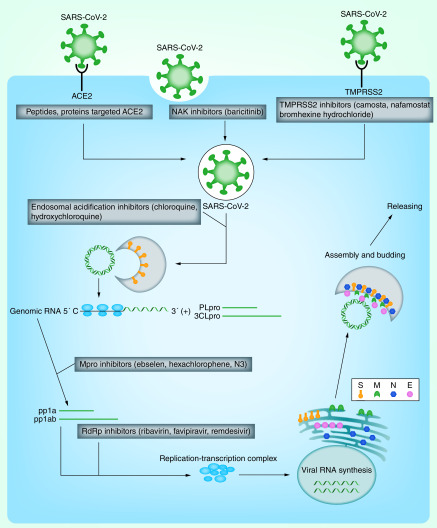
Potential drug targets and the corresponding inhibitors against SARS-CoV-2. E: Envelope; M: Membrane; N: Nucleocapsid; S: Spike.

SARS-CoV-2 contains a single-stranded, positive-sense RNA genome, the largest RNA virus identified so far, which is very similar to the virus that causes SARS and MERS. It belongs to the β-coronavirus genus and has 88% sequence homology with bat-SL-CoVZC45 and bat-SL-CoVZC21, and 50% sequence homology with MERS-CoV [2,3]. The SARS-CoV-2 genome contains at least ten open reading frames (ORFs). The first ORF (ORF1ab), which accounts for approximately two-thirds of the viral RNA, encodes 1–16 NSP at the consensus cleavage site. Important nonstructural proteins include RdRp, 3CLpro, PLpro. The rest ORFs, which account for the other third of the genome, encode four major structural proteins, spike (S), envelope (E), nucleocapsid (N), membrane (M) proteins and several proteins unknown functions. The SARS-CoV-2 replication cycle mainly includes virus entry, genome replication, assembly and germination of virions. Interruption of any stage of replication cycle is expected to become a potential strategy for the development of antiviral agents (Figure 1).

## Clinical trials of repositioning antiviral drugs for COVID-19

**Figure 2.**
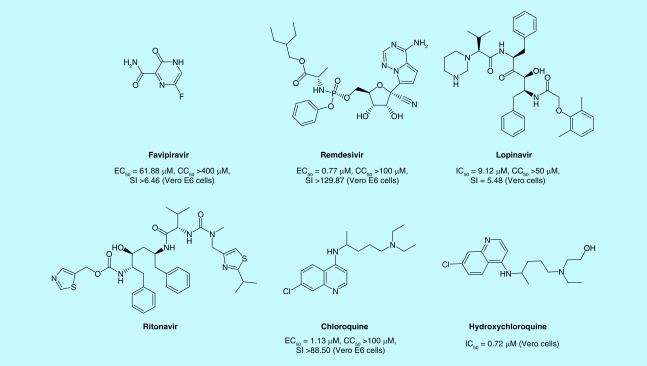
Structures and *in vitro* activities of repositioning antiviral drugs in clinic for SARS-CoV-2. CC_50_: 50% Cytotoxic concentration; EC_50_: Half maximal effective concentration; IC_50_: Half maximal inhibitory concentration; SI: Selectivity index.

Since the quick transmission of corona virus could be catastrophic for the entire world, developing new anti-SARS-CoV-2 drugs from scratch is impractical to face the pressing global challenge [4]. Drug repositioning is an emerging strategy because these drugs have known pharmacokinetic and pharmacodynamic properties, side effects and drug regimens [5]. RdRp and 3CLpro protease of SARS-CoV-2 share over 95% of sequence similarity with those of SARS-CoV and receptor-binding domain (RBD), a domain of S protein, was highly conserved between SARS-CoV and SARS-CoV-2 [6]. Thus, some anti-SARS-CoV, anti-MERS-CoV agents were chosen as a repositioning antiviral drug for COVID-19. Additionally, some known broad-spectrum antiviral drugs like nucleoside analogs and protease inhibitors were also chosen as a repositioning drug for COVID-19. Some drugs including favipiravir, remdesivir, lopinavir/ritonavir, chloroquine, hydroxychloroquine, ribavirin, darunavir, arb.i.d.ol, were clinically tested against COVID-19 infection (Figure 2). And they are recommended in guidelines of different countries (Table 1). Here we provide an update of latest results of clinical trials of repositioning antiviral drugs for COVID-19.

**Table 1.** Clinical trials of repositioning antiviral drugs for COVID-19.

Drug	Numbers of clinic trial	Status of clinic trial	Name of guideline	Ref.
Favipiravir	14	Recruiting: 3 Not yet recruiting: 9 Active, not recruiting: 1 Enrolling by invitation: 1	Treatment of novel coronavirus disease in Japan (first edition)	[54]
Remdesivir	21	Recruiting: 10 Not yet recruiting: 5 Available: 2 Enrolling by invitation: 1 Suspended: 1 Terminated: 1 Completed: 1	Expert recommendations on treating patients during SARS-CoV-2 epidemic (France) Clinical management of COVID-19: medical treatment (Spain) Antiviral therapy for patients with novel SARS-CoV-2 coronavirus infection (Greece)	[55–57]
Lopinavir/litonavir	53	Recruiting: 29 Not yet recruiting: 14 Active, not recruiting: 2 Enrolling by invitation: 3 Completed: 5	Diagnosis and treatment protocol for novel coronavirus pneumonia (trial version 7) Treatment of novel coronavirus disease in Japan (first edition) Expert recommendations for treating patients during the SARS-CoV-2 epidemic (France) Clinical management of COVID-19: medical treatment (Spain) Antiviral therapy for patients with novel SARS-CoV-2 coronavirus infection (Greece)	[54–58]
Chloroquine	57	Recruiting: 29 Not yet recruiting: 25 Enrolling by invitation: 3	Diagnosis and treatment protocol for novel coronavirus pneumonia (Trial Version 7) Expert recommendations on treating patients during SARS-CoV-2 epidemic (France) Drug treatment options for patients with COVID-19 (SARS-CoV-2 infection) (Netherlands) Antiviral therapy for patients with novel SARS-CoV-2 coronavirus infection (Greece)	[55,57–59]
Hydroxychloroq- uine	174	Recruiting: 80 Not yet recruiting: 72 Active, not recruiting: 4 Enrolling by invitation: 12 Completed: 3 Suspended: 3	Expert recommendations on treating patients during SARS- CoV-2 epidemic (France) Drug treatment options for patients with COVID-19 (SARS-CoV-2 infection) (The Netherlands) Antiviral therapy for patients with novel SARS-CoV-2 coronavirus infection (Greece)	[55,57,59]
Ribavirin	7	Recruiting: 3 Not yet recruiting: 2 Completed: 2	Diagnosis and treatment protocol for novel coronavirus pneumonia (trial version 7)	[58]
Darunavir	4	Recruiting: 2 Not yet recruiting: 1 Active, not recruiting: 1	Guidelines for the treatment and support management of patients with COVID-19 coronavirus infection (second edition; Italy)	[60]
Arb.i.d.ol	8	Recruiting: 3 Not yet recruiting: 3 Active, not recruiting: 1 Enrolling by invitation:1	Diagnosis and treatment protocol for novel coronavirus pneumonia (trial version 7) Expert recommendations on treating patients during SARS-CoV-2 epidemic (France)	[55,58]

### Favipiravir

Favipiravir (Figure 2) was developed by Toyama Chemical (Division of Fujifilm, Tokyo, Japan), and inhibits RdRp by structurally resembling the endogenous guanine. After oral administration, it is converted into the biologically active nucleoside triphosphate form. It was approved for marketing in Japan in March 2014 as an antiviral treatment for influenza A and B. Studies have shown that favipiravir has a specific inhibitory effect on SARS-CoV-2 (half maximal effective concentration [EC_50_] = 61.88 μM, 50% Cytotoxic concentration [CC_50_] >400 μM, and selectivity index [SI] >6.46) [7]. It possessed a similar anti-Ebola virus activity in vero E6 cells (EC_50_ = 67 μM), and effectively protected 100% of the mice subjected to an Ebola virus attack [8]. Thus, favipiravir is recommended for use in further research *in vivo*, at least eight trials having been already registered in the Chinese Clinical Trial Registry and at least 14 trials already registered on clinicaltrials.gov for SARS-CoV-2.

An open-label before-after controlled study (ChiCTR2000029600) enrolled 80 patients, with 35 in the favipiravir group and 45 in the control group [9]. The results showed that the median time for the negative conversion of viral nucleic acids in the favipiravir group was significantly shorter than it was in the participants in the control group (4 vs 11 days; p < 0.001). In terms of chest imaging, the rate of improvement was also significantly higher in the favipiravir group than that in the control group (91.43 vs 62.22%; p = 0.004).

Another multicenter, randomized, open, positive, parallel-controlled clinical study of the effectiveness of favipiravir compared with arb.i.d.ol in 240 patients was recently completed [10]. In the full analysis set (FAS) cohort of moderate patients with COVID-19, the clinical recovery rate at day 7 was 55.86% in the arb.i.d.ol group and 71.43% in the favipiravir group (p = 0.0199). Based on the above clinical trials results, favipiravir has been recommended to be included in the diagnosis and treatment programs in China.

### Remdesivir

Remdesivir (GS-5734, Figure 2) is a prodrug developed by Gilead Science, whose structure resembles adenosine. In vero E6 cells, the EC_50_ value of remdesivir for SARS-CoV-2 was 0.77 μM, the EC_90_ value was 1.76 μM and the SI was greater than 12 [7]. At least 21 trials have been already registered in clinicaltrials.gov for SARS-CoV-2.

On 1 February 2020, Holshue *et al.* reported that the first patient with SARS-CoV-2 in USA under deteriorating condition was compassionately treated with intravenous remdesivir, which improved the clinical symptoms, including a decrease in body temperature, no need for oxygen inhalation support and the return of oxygen saturation to 94–96% [11].

On 10 April 2020, Gilead Sciences Inc., published the first clinical result of compassionate-use remdesivir [12]. Of the 53 patients with severe COVID-19, 36 patients (68%) showed clinical improvement, 25 patients (47%) were discharged. whereas eight patients (15%) showed worsening and seven patients (13%) died. A total of 32 patients (60%) had side effects, 12 patients experienced serious side effects.

Two randomized, placebo-controlled trials (NCT04257656 and NCT04252664) of remdesivir were conducted in China. The clinical study (NCT04257656) is evaluating the efficacy and safety of remdesivir in patients hospitalized with severe COVID-19 and the other clinical study (NCT04252664) efficacy and safety of remdesivir in patients hospitalized with mild or moderate COVID-19. However limited by the patients recruited, the states of NCT04252664 and NCT04257656 were marked as suspended and terminated.

On 29 April 2020, the result of clinical trials (NCT04257656) was published [13]. Remdesivir was not associated with a difference in time to clinical improvement (hazard ratio for clinical improvement: 1.23; 95% CI: 0.87–1.75). In addition, remdesivir seemed to have little effect in reductions in SARS-CoV-2 RNA loads in upper respiratory tract or sputum specimens. In the subgroup analysis, receiving remdesivir treatment in the early stage, with symptom duration of 10 days or less, might be conducive to faster clinical improvement (hazard ratio for clinical improvement: 1.52; 95% CI: 0.95–2.43). But on the same day, Anthony S Fauci, the director of the National Institute of Allergy and Infectious Diseases (NIAID), declared that results from the global, placebo-controlled trial of remdesivir has reached the primary clinical end point. In this clinical study, the time to recover in remdesivir group is shorter than that in the control group (11 vs 15 days).

On 1 May 2020, Gilead Sciences Inc., announced that the US FDA had granted emergency use authorization (EUA) for the investigational antiviral remdesivir to treat COVID-19. On 8 May 2020, Japan approved remdesivir for use on severe COVID-19.

### Lopinavir/ritonavir

Lopinavir/ritonavir (Figure 2) is recommended as a second-line treatment of HIV. Probably inhibiting the action of 3CLpro, Lopinavir/ritonavir has been proved to be effective against SARS and MERS *in vitro* and *in vivo* [14]. Recent evidence suggests that lopinavir has antiviral activity against SARS-CoV-2 *in vitro* with an IC_50_ value of 9.12 μM [15]. At least 13 trials have been already registered in the Chinese Clinical Trial Registry and at least 53 trials already registered in clinicaltrials.gov for SARS-CoV-2.

The result of a randomized, controlled, open-label trial involving hospitalized adult patients with confirmed SARS-CoV-2 infection indicated that no benefit was observed with lopinavir/ritonavir treatment beyond standard care [16]. Lopinavir/ritonavir was not associated with benefit in hospitalized patients with COVID-19 ((hazard ratio for clinical improvement: 1.31; 95% CI: 0.95 to 1.80). Specifically, the difference in mortality between the lopinavir/ritonavir group and the control group failed to reach the statistically significant (19.2 vs 25.0%; difference: -5.8 percentage points; 95% CI, -17.3 to 5.7).

### Chloroquine

Chloroquine phosphate (Figure 2) has been commercialized as an antimalarial drug for more than 70 years. The *in vitro* anti-SARS-CoV-2 activity of chloroquine phosphate has been identified with an IC_50_ value of 1.13 μM and it was found to be effective in preventing replication of this virus [7]. Chloroquine phosphate could alkalise the phagolysosome, which hampers the low-pH-dependent steps of viral replication, including fusion and uncoating [17]. Chloroquine phosphate also could interfere with the glycosylation of ACE2 receptors, thus inhibiting the adsorption of SARS-CoV onto host cells [18]. Chloroquine phosphate is safe and side effects are generally mild and transitory. Based on the above results, at least 11 trials have been already registered in the Chinese Clinical Trial Registry and at least 57 trials already registered on clinicaltrials.gov for SARS-CoV-2.

In a recent publication, Gao *et al.* indicated that chloroquine phosphate was helpful in preventing the progression of COVID-19 and promoting a virus negative conversion in a multicenter clinical trial in China [19]. In this study, 120 patients with SARS-CoV-2 pneumonia were treated with chloroquine phosphate and 110 patients had undetectable viral RNA on the throat swab after the treatment, 81 patients were discharged. No serious adverse reactions were observed during the treatment. Based on these results, chloroquine phosphate was included in China's ‘Diagnosis and Treatment Protocol for COVID-19 (trial version 7)' followed by inclusion into the protocols of several other countries.

A parallel, double-masked, randomized, Phase IIB clinical was conducted to evaluate the safety and efficacy of chloroquine diphosphate in patients with severe COVID-19 [20]. 41 Patients received high-dosage chloroquine phosphate (i.e., 600 mg chloroquine phosphate twice daily for 10 days) and 41 patients received low-dosage chloroquine phosphate (i.e., 450 mg twice daily on day 1 and once daily for 4 days). Until day 13, 16 patients died in the high-dose group, whereas six patients died in the low-dose group. Simultaneously, QTc interval prolongation was more frequent in the high-dosage group than in the low-dosage group (18.9 vs 11.1%). Based on these results, high chloroquine phosphate dosage should not be recommended for critically sever COVID-19 patients because of its serious adverse effects, especially when taken concurrently with azithromycin and oseltamivir. These findings cannot be extrapolated to patients with nonsevere COVID-19.

In short-term use, chloroquine phosphate can prolong the QTc interval and induce arrhythmias. This is especially concerning in elderly patients with underlying heart disease who are at highest risk for COVID-19 [21]. Use of chloroquine phosphate should therefore be subject to strict rules. Further studies are needed to identify the optimal dose for COVID-19.

### Hydroxychloroquine

Hydroxychloroquine, an antimalarial agent, is now broadly used in autoimmune diseases such as lupus and rheumatoid arthritis. Yao *et al.* found that hydroxychloroquine (IC_50_ = 0.72 μM) was more effective than chloroquine (IC_50_ = 5.47 μM) *in vitro* [22]. Hydroxychloroquine has the same mechanism as chloroquine, but it is tolerated better, and its safety makes it the preferred drug for treating malaria and autoimmune diseases. Currently, at least eight trials have been already registered in the Chinese Clinical Trial Registry and at least 174 trials already registered on clinicaltrials.gov for SARS-CoV-2. Hydroxychloroquine is widely used, and its use is now permitted by the US FDA.

In a clinical trial with 36 patients, hydroxychloroquine sulfate showed a preliminary effect [23]. At day 6 of post-inclusion, 70% of hydroxychloroquine-treated patients were virologicaly cured compared with 12.5% in the control group (p = 0.001). At day 6 of post-inclusion, 100% of patients treated with hydroxychloroquine and azithromycin combination were virologicaly cured compared with 57.1% in patients treated with hydroxychloroquine only, and 12.5% in the control group (p < 0.001). Unfortunately, on 3 April 2020, International Society of Antimicrobial Chemotherapy (ISAC) declared the trial did “not meet the society's expected standard”.

Another prospective open label study of 11 people (seven men and four women) hospitalized with COVID-19 (10/11 with fever) at a single hospital in Paris, reported no benefits from using hydroxychloroquine combined with the antibiotic azithromycin were observed [24]. The treatment included hydroxychloroquine (600 mg/d for 10 days) and azithromycin (500 mg, day 1 and 250 mg, days 2 to 5). After 5 days, SARS-CoV-2 remained detectable by qualitative PCR in throat swabs in all participants. One patient died and two were transferred to intensive care units. One participant discontinued treatment after 4 days due to QT prolongation (from 405 ms to 460 and 470 ms).

Still, general concerns about the serious adverse reactions brought by hydroxychloroquine, including fulminant hepatic failure, and ventricular arrhythmias (especially administrated with azithromycin). On 24 April 2020, FDA issued a warning on the use of hydroxychloroquine and chloroquine for treating COVID-19. The warning is related to the potential for the drugs to prolong the QT interval, especially when the drugs are combined with the antibiotic azithromycin.

Several other trials, including a large multicenter trial in USA, are ongoing and hopefully will provide additional crucial information about the efficacy and safety of hydroxychloroquine.

### Multidrug combinations

The combination of multiple drugs is an important strategy against SARS-CoV-2 (Table 2). It is common to combine drugs with different modes of action or to interfere at different steps of the virus replication cycle to improve antiviral efficacy and reduce the likelihood of drug resistance. For example, the combination of endocytosis inhibitors and protein kinase inhibitors more effectively blocks viral invasion than a single inhibitor administered alone. According to Cohen *et al.* [25], an ideal treatment for SARS-CoV-2 infection is a combination of remdesivir and dendritic protein monoclonal antibodies. Many clinical studies based on this strategy have been carried out, including those with an RDRP inhibitor, such as favipiravir in tablet form, combined with endocytosis inhibitor chloroquine phosphate, in the treatment of COVID-19 (ChiCTR2000030987).

**Table 2.** Multi-drug combinations for COVID-19 in clinical trials.

Combination drug	Mechanism of action	Registered trials
Ribavirin + lopinavir/ritonavir + INF-α	Nucleoside inhibitor + protease inhibitor + regulates the activity of the immune system	ChiCTR2000029387, ChiCTR2000029573
Ribavirin + lopinavir/ritonavir + IFN-α	Nucleoside inhibitor + protease inhibitor + regulates the activity of the immune system	NCT04276688
Ribavirin + interferon	Nucleoside inhibitor + Regulates the activity of the immune system	ChiCTR2000030922
Favipiravir + chloroquine	Pyrazinecarboxamide derivative viral RNA polymerase inhibitor + heme polymerase inhibitor	ChiCTR2000030987
Favipiravir + tocilizumab	Pyrazinecarboxamide derivative viral RNA polymerase inhibitor + anti-human IL-6 receptor	ChiCTR2000030894, NCT04310228
Lopinavir/ritonavir + arb.i.d.ol	Protease inhibitor + hemagglutinin inhibitor	NCT04252885
Ganovo + ritonavir	Protease inhibitor	ChiCTR2000030472, ChiCTR2000030259, NCT04291729
Darunavir/cobicistat + thymosin a1; Lopinavir/ritonavir + thymosin a1	Protease inhibitor + stimulation of the development of precursor T cells	ChiCTR2000029541
Lopinavir/qitonavir + emtritabine/tenofovir alafenamide fumarate	Protease inhibitor + reverse transcriptase inhibitor	ChiCTR2000029468

Another important factor for the use of combined medications is the severity of the disease. Especially in patients with severe illness, the pathology is very complex and includes extensive immune dysregulation. The regimen may require antiviral drugs combined with immunomodulatory drugs. The combination of ribavirin and various types of interferon to enhance the innate antiviral response was the most commonly used in the treatment of patients with a coronavirus infection (such as SARS and MERS) [26–28]. A number of COVID-19 clinical studies based on this strategy are being conducted (ChiCTR2000029387 and ChiCTR2000030922). A combination of interferon-β and antiviral drugs, such as lopinavir/ritonavir, has also been recommended by clinical experts [29]. The *in vivo* and *in vitro* effects of remdesivir combined with IFN on MERS were better than those of lopinavir/ritonavir combined with IFN [30], which may be effective against SARS-CoV-2. Moreover, a current multicenter, randomized controlled clinical study of the antiviral drug favipiravir combined with the cytokine inhibitor tocilizumab in the treatment of COVID-19 is ongoing (ChiCTR2000030894 and NCT04310228).

## SARS-CoV-2 specific antiviral drugs for COVID-19

In the development of anti-SARS-CoV-2 drugs aimed at viral targets, a series of drug screenings have been performed targeted important proteins involved in various functions, such as pre-genomic recognition binding, endocytosis, membrane fusion, posttranscriptional modifications and replication, from which many potential anti-SARS-CoV-2 candidate compounds have been identified. In addition, some COVID-19 clinical studies have been conducted on these drugs, and some drugs have been used for emergency treatment in patients.

### Inhibitors targeting SARS-CoV-2 RdRp

Inhibitors targeting RdRp can incorporate into nascent viral RNA, and further inhibit the RdRp. This results in premature termination of the viral RNA chain and consequently halts the replication of the viral genome. In March 2020, Gao *et al.* successfully resolved the 3D spatial structure of the SARS-CoV-2 RdRp–nsp7–nsp8 complex (Protein Data Bank [PDB]: 6m71) [31]. Due to the crucial function of nsp12, the characterization of its structure in complex with its cofactors nsp7 and nsp8 provides atomic-level information to facilitate rational antiviral drug design and development.

**Figure 3.**
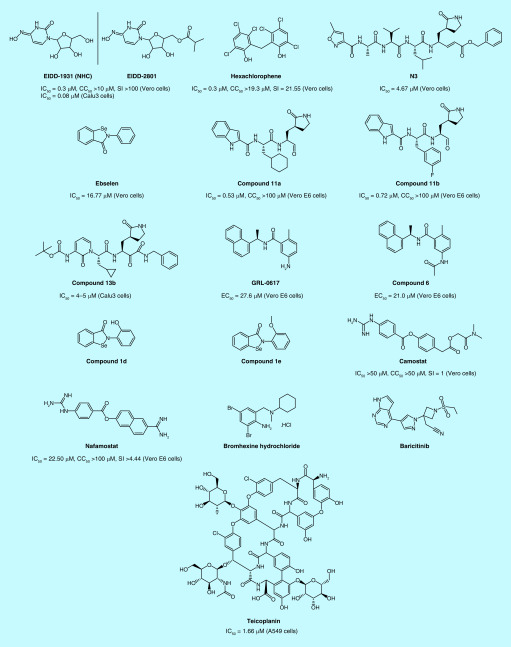
Structures and *in vitro* activities of SARS-CoV-2 specific antiviral drugs for SARS-CoV-2. CC_50_: 50% Cytotoxic concentration; EC_50_: Half maximal effective concentration; IC_50_: Half maximal inhibitory concentration; SI: Selectivity index.

EIDD-2801 (Figure 3), an isopropyl ester prodrug of the nucleoside analog EIDD-1931 (NHC, Figure 3), is hydrolyzed into the original drug NHC *in vivo*, which then interferes with viral RNA replication through prephosphorylation. NHC has demonstrated strong inhibition of SARS-CoV-2 activity in both Vero cells and Calu3 cells, with an IC_50_ value of 0.3 and 0.08 μM, respectively [32]. Molecular docking demonstrated that NHC and the N4 hydroxyl group on the cytidine ring form an additional hydrogen bond with the side chain of K545. In addition, cytidine bases also form an additional hydrogen bond with guanine bases. These extra hydrogen bond effects may be responsible for the high activity of NHC. At the same time, the main advantage of NHC is that it can avoid the cleavage of ExoN exonuclease and show strong inhibitory activity in mutant cell line resistant to remdesivir. Sheahan *et al.* demonstrated that EIDD-2801 (50 mg/kg, p.o., b.i.d.) could effectively decrease virus titers in mice infected with SARS and MERS models. Based on the excellent preclinical characteristics of EIDD-2801, the IND application of EIDD-2801 for the treatment of COVID-19 was approved by the FDA on 6 April 2020.

### Inhibitors targeting SARS-CoV-2 Mpro protein

Mpro protein, also known as the 3CLpro protein, is essential for proteolytic maturation of SARS-CoV-2. MPro cleaves the viral polyproteins, generating 12 non-structural proteins (Nsp4–Nsp16), which includes RdRp and helicase. The inhibition of MPro would prevent the virus from replication and therefore constitutes one of the potential anti-coronaviral strategies [33].

In March 2020, Jeon *et al.* [15] reported that hexachlorophene (Figure 3) could inhibit SARS-CoV-2 replication (IC_50_ = 0.90 μM, CC50 = 19.3 μM and SI = 21.55). As indicated by Liu *et al.* [34], hexachlorophene has inhibitory activity on the MPro protease with IC_50_ value of 5 μM, indicating its potential as a MPro inhibitor. On 9 April, 2020, the crystal structure of SARS-CoV-2 Mpro in complex with N3 (Figure 3), a Michael acceptor inhibitor, which can specifically inhibit multiple CoV Mpros from SARS-CoV and MERS-CoV at 2.1 Å resolution was reported [35]. Then researchers used Förster resonance energy transfer (FRET) assay to screen a library of approximately 10,000 compounds consisting of approved drugs, clinical trial drug candidates and natural products. Ebselen (Figure 3) has the strongest inhibition activity of Mpro with an IC_50_ value of 0.67 μM. Ebselen and N3 displayed inhibition against SARS-CoV-2 with an EC_50_ value of 4.67 and 16.77 μM, respectively. On 22 April, 2020, two lead compounds (**11a** and **11b**; Figure 3) targeting SARS-CoV-2 Mpro were designed and synthesized [36]. Both **11a** and **11b** exhibited high SARS-CoV-2 Mpro inhibition activity with an IC_50_ value of 0.053 ± 0.005 μM and 0.040 ± 0.002 μM, respectively and exhibited potent anti-SARS-CoV-2 infection activity with an EC_50_ value of 0.53 ± 0.01 μM and 0.72 ± 0.09 μM using plaque-reduction assay, respectively. X-ray crystal structures of SARS-CoV-2 Mpro in complex with **11a** or **11b**, both determined at 1.5 Å resolution, showed that aldehyde groups of **11a** and **11b** are covalently bound to Cys145 of Mpro. On 24 April 2020, x-ray structures of the unliganded SARS-CoV-2 Mpro at 1.75 Å resolution and its complex with an a-ketoamide inhibitor were reported [37]. Compound **13b** (Figure 3) inhibits the purified recombinant SARS-CoV-2 Mpro with an IC_50_ value of 0.67 ± 0.18 μM and inhibits SARS-CoV-2 replication in human Calu-3 lung cells with an EC_50_ value of 4–5 μM. Structural information from these related proteins could be vital in furthering our understanding of SARS-CoV-2 and in discovery and development of novel specific anti-SARS-CoV-2 drugs.

### Inhibitors targeting SARS-CoV-2 PLpro

PLpro is responsible for processing three cleavage sites of the viral polyprotein to release mature non-structual proteins 1, 2 and 3, which is essential for correcting virus replication. The PLpro is also responsible for suppression of host innate immune responses upon a deubiquitinase and deISGylating activity. Based on the above function, PLpro represents a promising target for the antiviral drugs.

On 27 January 2020, Wu *et al.* [38] firstly performed a docking study to discover the potential drugs targeted SARS-CoV-2 PLpro. In this study, they demonstrated 33 virtual potential inhibitors of SARS-CoV-2 PLpro, such as ribavirin, valganciclovir and thymidine, chloramphenicol, cephadol, tigecycline and levoperazine. On 20 February 2020, Arya *et al.* [39] carried out homology modeling by using the SARS-CoV PLpro crystal structure (PDB ID: 3E9S) as a template. Based on the homology model and the following docking study, 16 FDA approved drugs, including chloroquine and formoterol, were screened out with significant affinity. On 19 May 2020, Freitas *et al.* [40] reported that naphthalene based PLpro inhibitors seemed to be active against SARS-CoV-2 PLpro. By screening the previously developed SARS-CoV naphthalene based PLpro inhibitors, they found that compound GRL-0617 (Figure 3) and compound **6** (Figure 3) could inhibit the PLpro of SARS-CoV-2, with an IC_50_ values of 2.4 and 5.0 μM respectively. GRL-0617 and compound **6** inhibits SARS-CoV-2 replication in Vero E6 cells with an EC_50_ of 27.6 and 21.0 μM. On 19 May 2020, Ewelina *et al.* [41] reported a series of selenium-containing compounds which could inhibit SARS-CoV-2 PLpro *in vitro*. Among them, compound **1d** and **1e** (Figure 3) were the most potent with an IC_50_ of 236 ± 107 nM and 256 ± 35 nM. But the activity against SARS-CoV-2 was not reported.

### TMPRSS2 inhibitor

On 16 April 2020, Hoffmann *et al*. reported that SARS-CoV-2 used ACE2 for entry and the serine protease TMPRSS2 for S protein priming [42]. They also found that the serine protease inhibitor camostat (Figure 3) mesylate could block SARS-CoV-2 infection of lung cells. Camostat has been approved in Japan for human use, but for an unrelated indication. TMPRSS2 inhibitors with potentially increased antiviral activity could thus be considered for off-label treatment of SARS-CoV-2-infected patients. On 18 March 2020, scientists of Tokyo University reported that nafamostat (Figure 3), another TMPRSS2 inhibitor marketed for the treatment of pancreatitis, could inhibit membrane fusion at a concentration of 1–10 nm, preventing SARS-CoV-2 from invading into primary target cells [43]. The activity of nafamostat was tenfold potent than camostat, and it was also speculated that the blood concentration of nafamostat may exceed the membrane fusion inhibition concentration after administration. Therefore, it was expected to be effective for the treatment of COVID-19. Wang *et al.* also found that nafamostat had specific inhibitory activity on SARS-CoV-2 (IC_50_ = 22.50 μM, CC50 >100 μM and SI >4.44) [7]. Lucas *et al.* reported five effective TMPRSS2 inhibitors, including bromhexine hydrochloride (Figure 3) with the lowest IC_50_ value of 0.75 μM toward TMPRSS2. Currently, bromhexine hydrochloride was clinically tested against COVID-19 infection [44].

### Numb-associated kinase inhibitors

Richardson *et al.* found that AAK1 inhibitors might interrupt the passage of the virus into cells and also the intracellular assembly of virus particles [45,46]. Among 378 AAK1 inhibitors, baricitinib (Figure 3), binding the cyclin G-associated kinase, another regulator of endocytosis, has been shown to inhibit viral infection of cells through the inhibition of AAK1. Baricitinib could reduce both the viral entry and the inflammation in patients. It has high affinity to the key regulator of clathrin-mediated endocytosis, AAK1. Baricitinib, developed by Incyte and Eli Lilly, have been already used to treat rheumatoid arthritis. The drug has been registered in clinical trials for COVID-19 (NCT04321993).

### Anti-infective drugs with anti-SARS-CoV-2 activity

Teicoplanin (Figure 3), a glycopeptide antibiotic routinely used to treat bacterial infections, could be used in the therapeutic arsenal against COVID-19. It could prevent the release of viral RNA by inhibiting the low-pH cleavage of the viral spike protein in the late endosomes. Teicoplanin shows inhibitory activity against SARS-CoV-2 with an EC_50_ value of 1.66 μM, which is much lower than the concentration reached in human blood (8.78 μM for a daily dose of 400 mg) [47]. These preliminary results now need to be confirmed in a randomized clinical trial.

## Challenges

### Insufficient understanding of the SARS-CoV-2

First, the scientific understanding of the virus is insufficient. COVID-19 is a new emerging infectious disease that is not exactly the same as SARS or MERS. With the understanding of the virus, including its clinical symptoms, manifestation and treatment being constantly updated, researchers may also change the strategies of drug development, which hampers the development process of drugs being used for the treatment of COVID-19. In addition, the severity of the viral infection, especially the mortality rate, is not well analyzed, making it difficult to evaluate the risk/benefit ratio. In contrast to those exposed to SARS, many patients infected with SARS-CoV-2 have no symptoms; therefore, the causes of severe illness are unclear.

Secondly, the continuous variation of the virus hinders drug development. As a single-stranded envelope virus, SARS-CoV-2 is relatively prone to gene mutation and recombination to adapt to different hosts. Fortunately, the current research suggests that the virus has not mutated significantly [48–50]. However, the L and S subtypes have evolved since the outbreak [51]; concurrently, a deletion mutation of a large fragment consisting of 382 nucleotides has occurred (Δ382). Virus variations may lead to drug resistance quickly, which means that drug development will become more difficult. For example, the broad-spectrum antiviral drug Kaletra shows poor efficacy for SARS-CoV-2 infection.

Moreover, the development of anti-coronavirus (anti-CoV) drugs has long been hindered by the unique nsp14-ExoN of CoVs. The nsp14 MTase domain possesses methyltransferase activity and is critical for capping viral mRNA. nsp14-ExoN makes CoVs naturally resistant to many nucleoside analogs, such as ribavirin and 5-fluorouracil.

### Lack of proper drug evaluation model

First, there is a lack of a preclinical COVID-19 infected animal model. The greatest challenge in developing new drugs is the lack of a preclinical evaluation system. Therefore, it is impossible to effectively distinguish which candidate compounds are the most likely to become effective drugs. Currently, several agencies are scrambling to build animal models [52,53]. The Chinese team first discovered that macaques and mice have *ACE2* genes suitable for SARS-CoV-2 studies; the Beijing Union Medical College and Wuhan Virus Institute in China have successfully infected human ACE2 transgenic mice with SARS-CoV-2, which showed some infection symptoms; Guangzhou Medical University have established nontransgenic COVID-19 infected pneumonia mouse models; Wuhan Virus Institute has preliminarily established primate models. Through CT scanning, they observed some symptoms, such as the viral load. The Australian Animal Health Lab in Geelong discovered that ferrets are sensitive to SARS-CoV-2 infection and the physiological lung structure of ferrets and humans are similar. However, in general, the present SARS-CoV-2 animal models need to be further improved. Currently, mice exhibit only mild symptoms after infection, and new models should be developed to better mimic severe cases.

Secondly, high-quality clinical trials are difficult to organize. The biggest obstacle is the recruitment of suitable patients even with effective clinical drugs in hand. Since the outbreak, more than 200 clinical trials have been carried out in China alone, and the rationality of some clinical studies needs to be explored to ensure that clinical resources are not wasted. In addition, conducting placebo-controlled trials with patients suffering with severe symptoms needs ethical considerations.

## Future perspective

SARS-COV-2 has been widely spread all over the world and has caused a serious real threat to human health. So far, there are no effective means to control the COVID-19 epidemic. Scientists around the world must work together to develop effective drugs and vaccines to reduce the impact on the global health system and human life. To alleviate the pressure of global pandemic situation, the new use of old drugs has become the main method for the treatment of COVID-19 in the short term. Many potential drugs are under clinical research, which includes RdRp inhibitor remdesivir, the host-specific SARS-CoV-2 inhibitors chloroquine, hydroxychloroquine and multiple drug combinations. We hope that the efficacy will be finally verified in randomized, double-blind clinical studies. In the long term, a globally accessible high-throughput evaluation system should be established. Once a new virus arrives, scientists could quickly assess the success rate of existing drugs to avoid the chaos of performing hundreds of clinical trials. The long-term drug development goal should be developing therapeutic drugs that can produce broad-spectrum therapeutic effects on different coronaviruses. This article comprehensively introduces the current challenges of COVID-19 therapeutic drug development, proposes corresponding strategies, combs the potential therapeutic targets of SARS-COV-2 and corresponding drugs.

**Executive summary**

- SARS-CoV-2 emerged in December 2019 and COVID-19 caused by SARS-CoV-2 was declared a global pandemic by the World health Organization (WHO).
- Only remdesivir has been approved to treat COVID-19. So the development of anti-SARS-CoV-2 drugs has become the most important work.

**Structure of SARS-CoV-2 & potential drug targets**

- SARS-CoV-2 contains a single-stranded, positive-sense RNA genome, which is the largest RNA virus identified so far.
- Important non-structural proteins include RdRp, 3CLpro, helicase and PLpro, major structural proteins include spike, envelope, nucleocapsid and membrane proteins.

**Clinical trials of repositioning antiviral drugs for COVID-19**

- Drug repositioning, an effective drug discovery strategy from existing drugs, could significantly shorten the time and reduce the cost. The advantage is that pharmacokinetic properties, dosages, potential efficacy and side effects of the marketed drug are relatively clear.
- Successful repositioning antiviral drugs, including favipiravir, remdesivir, lopinavir/ritonavir, chloroquine and hydroxychloroquine, are currently under clinical trials to verify efficacy. They are also recommended in guidelines of different countries.

**SARS-CoV-2 specific antiviral drugs for COVID-19**

- The structures of many important viral target proteins and host target proteins, including RdRp, Mpro and so on were constantly reported, which greatly promoted the ability to design targeted drugs.
- Based on the crystal structures of target proteins, effective compounds, such as EIDD-2801, compound **11a** and **11b**, displayed potent activity *in vitro*. Some of them have already entered clinical research.

**Challenges**

- The scientific understanding of the virus is insufficient, including the its clinical symptoms, manifestation and so on. Worse still, SARS-CoV-2 mutate constantly, which further hinders the drug development.
- Another challenge in developing new drugs is the lack of a preclinical evaluation system. The present SARS-CoV-2 animal models need to be further improved. High-quality clinical trials are also difficult to organize.
